# Cardiovascular Supportive Therapies for Neonates With Asphyxia — A Literature Review of Pre-clinical and Clinical Studies

**DOI:** 10.3389/fped.2018.00363

**Published:** 2018-12-10

**Authors:** Chloe Joynt, Po-Yin Cheung

**Affiliations:** ^1^Department of Pediatrics, University of Alberta, Edmonton, AB, Canada; ^2^Department of Pharmacology, University of Alberta, Edmonton, AB, Canada; ^3^Centre for the Study of Asphyxia and Resuscitation, Edmonton, AB, Canada

**Keywords:** newborn, asphyxia, inotropes, catecholamines, hemodynamics

## Abstract

Asphyxiated neonates often have hypotension, shock, and poor tissue perfusion. Various “inotropic” medications are used to provide cardiovascular support to improve the blood pressure and to treat shock. However, there is incomplete literature on the examination of hemodynamic effects of these medications in asphyxiated neonates, especially in the realm of clinical studies (mostly in late preterm or term populations). Although the extrapolation of findings from animal studies and other clinical populations such as children and adults require caution, it seems appropriate that findings from carefully conducted pre-clinical studies are important in answering some of the fundamental knowledge gaps. Based on a literature search, this review discusses the current available information, from both clinical studies and animal models of neonatal asphyxia, on common medications used to provide hemodynamic support including dopamine, dobutamine, epinephrine, milrinone, norepinephrine, vasopressin, levosimendan, and hydrocortisone.

## Introduction

Asphyxia is a clinico-pathological condition that is caused by a hypoxic-ischemic insult resulting in dysfunction of one or more organ systems in over 80% of asphyxiated neonates (1, 2). Asphyxiated neonates may have a decreased cardiac output (CO) state with ventricular myocardial dysfunction, decreased left ventricular preload secondary to pulmonary hypertension and/or decreased ability to regulate vessel tone (vasoplegia). The critically ill neonate may have hypoxia-ischemia of vital organs including the brain, intestine, kidney and lung (2–4). Therefore, asphyxiated neonates have significant mortality and long-term morbidity including physical and mental disability (2, 5, 6).

Cardiovascular support is often provided using a myriad of medications to treat the complex and heterogeneous etiologies of hemodynamic compromise or instability in asphyxiated neonates. Most commonly, “antihypotensive” treatments are administered when the infant develops low blood pressure (BP) or signs of low CO. However, the challenge for clinicians caring for asphyxiated neonates is to understand the pathophysiology of hemodynamic disturbances and then provide an individualized therapy to the patient. It is important to be cognizant of a hemodynamic state that evolves through feto-neonatal transition in the course of an asphyxiating disease and recovery, as well as the interaction between hemodynamics and concurrent treatments including respiratory state and positive pressure ventilation, and therapeutic hypothermia (TH).

Many clinicians assume that BP and systemic perfusion are proportionally related. However, this assumption requires validation as BP, vascular resistance and CO can be independent yet interactive factors for systemic and regional perfusion. Evaluation of cardiac performance and regional tissue oxygenation provide important information to understand systemic and regional hemodynamic state. Evaluation tools include echocardiographic examination, near-infrared spectroscopy, in addition to conventional markers such as acid-base balance, plasma lactate and urine output. Functional echocardiography may also provide point-of-care information regarding the ventricular function, shunting and volume status (7). The clinical application of near infrared spectroscopy has gained popularity for the real-time measurement of regional tissue perfusion and oxygenation trends in recent years (8).

While many of the neonatal studies of hypotension and treatments highlight that practices are variable across countries and heterogeneity of patients, the vast majority focus on the premature population but not asphyxiated neonates (9, 10). The study by Burns et al. reported 41% of those babies receiving cardiovascular supportive drugs were born at term with a variety of pathological conditions including necrotising enterocolitis, pulmonary hypertension (PHT), birth asphyxia, surgery, sepsis, and cardiac diseases (10). Although the use of cardiovascular supportive drugs was associated with increased mortality, this finding was not observed in the TH group (10)

In this review, we searched the literature of clinical (Table 1) and pre-clinical animal (Table 2) studies regarding the hemodynamic effects of dopamine, dobutamine, epinephrine, milrinone, norepinephrine, vasopressin, levosimendan, and hydrocortisone administration in newborn or infantile subjects with asphyxia, hypoxia-reoxygenation, or cardiac surgery. We will discuss the hemodynamic effects of various cardiovascular supportive therapies in asphyxiated neonates following resuscitation. Other therapies including methylene blue (an inhibitor of nitric oxide-cGMP pathway), calcium (in hypocalcemic conditions), naloxone (an antagonist of opioids), and triiodothyronine (an inotropic hormone) will not be discussed because the clinical experience in neonatal asphyxia or hypoxia-reoxygenation is anecdotal.

**Table 1:** Relevant clinical studies on the hemodynamic effects of cardiovascular medications in term or near-term neonates with asphyxia and other conditions.

**Publication**	**Clinical population**	**Medication studied**	**Significant findings**	**Study design**
DiSessa et al. (11)	14 severely asphyxiated neonates	Dopamine 2.5 mcg/kg/min vs. placebo	Increased cardiac performance (echocardiography) and systolic blood pressure	Double-blind, randomized trial
Walther FJ et al. (12)	6 neonates with severe asphyxia	Dopamine 4–10 mcg/kg/min	Increased blood pressure and heart rate; improved left ventricular myocardial dysfunction (echocardiography)	Observational study
Devictor et al. (13)	6 neonates with severe asphyxia	Dobutamine 10 mcg/kg/min	Increased cardiac output, heart rate and aortic blood flow velocity with modest effect in blood pressure	Observational study
Chang et al. (14)	10 neonates after cardiac surgery	Milrinone 0.5 mcg/kg/min after loading dose of 50 mcg/kg	Improved cardiac index with reduced systemic and pulmonary arterial pressures and respective vascular resistances	Prospective cohort study
Hoffman et al. (15)	238 infants and children after cardiac surgery	Milrinone 0.25, 0.5, and 0.75 mcg/kg/min vs. placebo	High-dose milrinone reduced the risk of low cardiac output syndrome	Double-blind, randomized trial
Li et al. (16)	13 neonates after cardiac surgery	Dopamine 5 mcg/kg/min	Increased heart rate, rate-pressure product and systemic oxygen consumption	Prospective cohort study
McNamara et al. (17)	9 neonates with severe persistent pulmonary hypertension of newborn	Milrinone 0.33 mcg/kg/min (0.33–0.99 mcg/kg/min)	Improved (decreased) heart rate; reduced oxygenation index and inhaled nitric oxide dose	Retrospective Observational study
Lechner et al. (18)	17 neonates with vasopressor-resistant hypotension after cardiac surgery	Vasopressin 0.0001–0.0003 (0.00005–0.001) U/kg/min	Increased blood pressure and decreased dosage of traditional vasopressors	Prospective cohort study
Tourneux et al. (19)	22 neonates with persistent septic shock	Norepinephrine 0.5 ± 0.4 mcg/kg/min	Increased arterial blood pressure and urine output with decreased lactate	Prospective observational study
Tourneuxet al. (20)	18 neonates with PPHN and cardiac dysfunction	Norepinephrine 0.5 ± 0.4 mcg/kg/min	Increased systemic blood pressure, cardiac output and pulmonary artery blood flow (echocardiography); decreased pulmonary/systemic pressure ratio and oxygen need	Prospective observational study
Bravo et al. (21)	5 neonates after cardiac surgery with low cardiac output syndrome refractory to conventional therapies	Levosimendan 0.1–0.2 mcg/kg/min	Improved cerebral and systemic perfusion and oxygenation (near infra-red spectroscopy)	Prospective observational study
De Carolis et al. (22)	2 neonates with heart failure and pulmonary hypertension	Levosimendan 0.1–0.2 mcg/kg/min		Prospective observational study
Ricci et al. (23)	63 neonates after cardiac surgery	Levosimendan 0.1 mcg/kg/min vs. placebo	Reduced the risk of low cardiac output syndrome; lowered inotropic score and lactate level	Double-blind, randomized trial
Alten et al. (24)	37 neonates after cardiac surgery	Early post bypass Vasopressin 0.0003 U/kg/min (0.0008–0.001) vs. no vasopressin	Lower inotropic score and fluid resuscitation	Retrospective cohort observational study
McNamara et al. (25)	11 neonates with severe persistent pulmonary hypertension of newborn	Milrinone 0.33–0.99 mcg/kg/min after loading dose of 50 mcg/kg	Improved cardiac output; reduced pulmonary artery pressure; reduced oxygenation index and inhaled nitric oxide dose	Observational study
Mohamed et al. (26)	10 neonates with severe persistent pulmonary hypertension of newborn	Vasopressin 0.0002 ± 0.0002 U/kg/min	Increased blood pressure and urine output; improved oxygenation index	Retrospective observational study
Oualha M et al. (27)	39 children after cardiac surgery	Epinephrine 0.01–0.23 mcg/kg/min with milrinone	Increased blood pressure, heart rate, plasma glucose and lactate	Prospective observational study
Bianchi et al. (28)	17 neonates prior to cardiac surgery	Milrinone 0.75 mcg/kg/min	Increased cardiac output, superior mesenteric and cerebral mean velocities	Prospective observational study
James et al. (29)	17 neonates with severe persistent pulmonary hypertension of newborn	Milrinone 0.5–0.7 mcg/kg/min	Increased cardiac output; reduced inhaled nitric oxide dose and oxygen requirement	Retrospective observational study
Giaccone et al. (30)	6 neonates with severe persistent pulmonary hypertension of newborn	Milrinone 0.2 and 0.5 mcg/kg/min preceded by 20 or 50 mcg/kg bolus respectively	Variable response in oxygenation index which improved with time	Multi-center randomized open label pilot study

**Table 2:** Relevant intact animal studies on the hemodynamic effects of cardiovascular medications in neonatal asphyxia.

**Publication**	**Cardiovascular medication(s)**	**Research model and design**	**Significant findings**	**Remarks**
Walker et al. (31)	Vasopressin 0.004 U/kg/min iv	Sprague-Dawley adult rats; Conscious, unrestrained; Normoxia and hypoxia	Increased SAP; decreased PAP (hypoxia>normoxia), CO and HR	
Caspi et al. (32)	Epinephrine 0.5 and 2.0 mcg/kg/min	Newborn piglets; Acutely instrumented; Normoxia	Increased end-systolic elastance in high-dose followed by a decrease after 2 h infusion, along with an increase in left ventricular volume elasricity	Associated with sarcolemmal rupture and mitochondrial calcium granule deposition
Barrington KJ et al. (33)	Dopamine 2–16 mcg/kg/min vs. Epinephrine 0.2–1.6 mcg/kg/min	Newborn piglets; Acutely instrumented; Normoxia and hypoxia; Prospective, randomized, blind, cross-over study	Both drugs increased CO. Epinephrine in normoxia: increased SAP with reduced PAP/SAP ratio. Epinephrine with hypoxia: increased SAP, reduced PAP, with reduced PAP/SAP ratio	
Ferrara JJ et al. (34)	Dopamine 5–15 mcg/kg/min vs. Dobutamine 5–15 mcg/kg/min	Newborn piglets; term and preterm (90% gestation); Acutely instrumented; Normoxia	Both drugs increased SAP at 15 ug/kg/min in term animals; Dobutamine increased heart and brain blood flow but decreased small intestinal blood flow. Dopamine increased heart and small intestinal blood flows	
Cheung et al. (35)	Dobutamine 5–50 mcg/kg/min × 20 min and 10 mcg/kg/min × 1 h	Newborn piglets; Chronically instrumented; Normoxia; Prospective, randomized (dose) study	Dose-dependent increases in CO; no effect on mesenteric and renal blood flows; increased PAP/SAP ratio; prolonged infusion at 10 ug/kg/min increased stroke volume	
Penny et al. (36)	Dobutamine 1–40 mcg/kg/min	Newborn lambs; Acutely instrumented; Normoxia	Increased systemic oxygen delivery and consumption	Multiple adrenoceptors activation
Cheung et al. (37)	Dopamine 2–32 mcg/kg/min vs. Epinephrine 0.2–3.2 mcg/kg/min × 1 h	Newborn piglets; Acutely instrumented; Hypoxia; Prospective, randomized (dose) study	Epinephrine increased CO Dopamine increased PAP/SAP ratio, portal venous blood flow and oxygen delivery	
Al-Salam et al. (38)	Dobutamine 5–20 mcg/kg/min × 2 h	Newborn piglets; Acutely instrumented; Hypoxia-reoxygenation	High-dose dobutamine increased CO and stroke volume without effect on SAP, PAP and regional (carotid, mesenteric and renal) blood flows	
Cheung et al. (39)	Epinephrine 1 mcg/kg/min vs. Epinephrine 0.2 mcg/kg/min + Dopamine 10 mcg/kg/min × 2 h	Newborn piglets; Acutely instrumented; Hypoxia-reoxygenation; Prospective, randomized, blind study	Both regimens similarly improved CO and SAP; no changes in regional (carotid, mesenteric and renal) blood flows	Study of combined catecholamines vs. monotherapy
Obaid et al. (40)	Epinephrine 0.3–1.5 mcg/kg/min vs. Dopamine 10-25 mcg/kg/min × 2 h	Newborn piglets; Acutely instrumented; Hypoxia-reoxygenation; Prospective, randomized, blind study	Epinephrine increased CO and SAP with reduced PAP/SAP ratio. Modest effects with dopamine. Both regimens similarly increased regional (carotid and mesenteric, but not renal) blood flows	SAP-targeted study protocol
Joynt et al. (41)	Milrinone 0.25–0.75 mcg/kg/min × 2 h	Newborn piglets; Acutely instrumented; Hypoxia-reoxygenation; Prospective, randomized, blind study	Dose-response effect of milrinone in CO; no effect on SAP and PAP. High-dose milrinone increased carotid and mesenteric blood flow and oxygen delivery.	Fluid bolus prior to infusion; milrinone prevented aggravated pulmonary hypertension
Joynt et al. (42)	Dobutamine 20 mcg/kg/min vs. Epinephrine 0.5 mcg/kg/min vs. Milrinone 0.75 mcg/kg/min × 2 h	Newborn piglets; Acutely instrumented; Hypoxia-reoxygenation; Prospective, randomized, blind study	All regimens similarly increased cardiac output, stroke volume, carotid and mesenteric but not renal blood flows; Dobutamine and epinephrine increased SAP; Milrinone decreased renal vascular resistance	No aggravation of pulmonary hypertension
Ichikawa et al. (43)	Milrinone 1, 10 mg/kg ip	Sprague-Dawley newborn rats; Conscious, unrestrained; Normoxia	Milrinone dilated ductus arteriosus	
Esch et al. (44)	Levosimendan 0.1, 0.2 mcg/kg/min × 2 h	Newborn piglets; Acutely instrumented; Hypoxia-reoxygenation	Both doses increased CO, HR and decreased systemic vascular resistance; low-dose increased PAP; no effects on regional blood flows	
Cheung et al. (45)	Vasopressin 0.005–0.02 U/kg/h × 2 h	Newborn piglets; Acutely instrumented; Hypoxia-reoxygenation	Dose-dependently increased SAP with no effects on CO and regional blood flows	Showed a baro-specific action
Pelletier et al. (46)	Vasopressin 0.01 U/kg/h × 3.5 h vs. Dobutamine 20 mcg/kg/min × 2 h	Newborn piglets; Acutely instrumented; Hypoxia-reoxygenation; Prospective, randomized, blind study	Both regimens increased CO and mesenteric blood flow	Early administration of vasopressin prior to severe cardiogenic shock during recovery
Drury et al. (47)	Dopamine 4–40 mcg/kg/min vs. saline	Fetal sheep; Near-term (85% gestation); Chronically instrumented; Asphyxia by umbilical cord compression; Prospective, randomized, study	Dopamine infusion was associated with a marked but transient increase in SAP followed by terminal hypotension. No effect in carotid blood flow,	SAP-targeted study protocol; Fetal asphyxia model.
Manouchehri et al. (48)	Dopamine 10 mcg/kg/min + epinephrine 0.2 mcg/kg/min vs. Dopamine 20 mcg/kg/min × 2 h	Newborn piglets; Acutely instrumented; Hypoxia-reoxygenation; Prospective, randomized, blind study	Both regimens similarly increased CO, SAP, carotid and mesenteric blood flow, but not PAP. Dopamine + epinephrine decreased PAP/SAP ratio. High-dose dopamine decreased mesenteric vascular resistance	Study of combined catecholamines vs. monotherapy
Eiby et al. (49)	Dopamine 10, 20 ug/kg/min vs. dobutamine 10, 20 mcg/kg/min	Newborn piglets; term and preterm (90% gestation); Acutely instrumented; Normoxia	Both increased SAP and HR but not CO nor cerebral blood flow	Less responses in preterm vs. term piglets
Eriksen et al. (50)	Dopamine 10, 25, 40 mcg/kg/min	Newborn piglets; Acutely instrumented; Hypotension; Prospective, randomized (dose) study	Dopamine did not impair cerebral autoregulation	Dopamine tended to improve cerebral autoregulation at low blood pressures
Mielgoet al. (51)	Dobutamine* 10–15 and 15–20 mcg/kg/min × 30 min	Newborn piglets; Acutely instrumented; Hypoxia-reoxygenation; Prospective, randomized, blind, cross-over study	Both doses increased HR and CO but not stroke volume nor SAP; 10-15 ug/kg/min dobutamine increased oxygen consumption	*A new pediatric dobutamine formulation

*SAP and PAP, systemic and pulmonary arterial pressure, respectively; CO, cardiac output; HR, heart rate*.

## Variables in Hypotension or Low Perfusion States in Asphyxiated Neonates

In comparison with the heart of older children, the basal contractile state is higher and compliance is lower in the neonatal heart,(52) which also has a higher resting ventricular output per kilogram of body weight than that of adults (53). In addition, the neonatal cardiomyocytes' excitation-contraction coupling and transverse tubular framework is not yet fully developed and not overly resilient to a hypoxic insult (54). Thus, in the neonatal heart, reserves for contractility, ventricular output and energy are reduced with compromised capability in response to stressors.

In addition to the transitional and neonatal physiology including fetal shunts, asphyxiated neonates have altered cardiac and vascular functions that can result in hypotension^1^ and/or low CO^1^ or perfusion states. Furthermore, iatrogenic effects of concurrent treatments, such as TH and rewarming as well as ventilation can also contribute to the disruption of the hemodynamic homeostasis. Upon the resuscitation of asphyxiated neonates, reactive oxygen species are produced during reoxygenation and reperfusion. This can lead to adverse effects including plasma membrane disruption, mitochondrial dysfunction and apoptosis (55–59).

In asphyxiated neonates, attenuated parasympathetic and increased sympathetic nervous activities play a significant role in the hemodynamic changes with initial tachycardia and increased (or normalized) BP during asphyxia and after the resuscitation (60, 61). Following the initial recovery, cardiac dysfunction develops including reduced myocardial contractility and passive dilation, which often occur in conjunction with systemic hypotension and PHT (3, 62, 63). PHT of asphyxiated neonates has a multitude of effects on the compromised myocardium including decreased systemic blood flow, increased right ventricular afterload and stress, increased cardiac transmural pressure with decreased perfusion in the subendocardial layer, and aggravated systemic hypoxemia with right-to-left shunting across a patent ductus arteriosus and foraman ovale (64–66). With poor perfusion or CO, acidosis can develop, which can further aggravate PHT leading to increasing cardiac afterload and additive stress to a dysfunctional ventricle (63, 67).

Asphyxiated neonates may have vasomotor dysregulation even to the point of vasomotor paresis (vasoplegia) which may impair autoregulation or affect the redistribution of regional blood flow and perfusion (68, 69). The relationship between BP, CO, and organ perfusion may be altered with the interplay between BP-dependency and flow-dependency in the regulation of tissue perfusion and oxygenation as has been shown in the brain and gut (70–76).

When a decision to treat is made, it is imperative to consider the history of the baby and their antenatal and postnatal course, the dynamic state of the cardiovascular compromise and the contribution of cardiac, pulmonary, and vascular dynamics that may vary for each neonate. Clinical parameters should be put into context with the information available be it clinical, laboratory, echocardiographic or regional oxygenation as indicated by near infrared spectroscopy (77). In addition, the concomitant effects of treatments for asphyxia including TH, rewarming, sedation, ventilation, anticonvulsants, to name a few, should be considered in treatment decisions as to whether to initiate cardiovascular supportive treatment and in antihypotensive agent.

During TH, the heart rate falls and systemic vascular resistance may increase (54). Studies have shown that CO in cooled neonates is approximately 2/3 of their normothermic baseline (78). Although myocardial contractility may be enhanced during TH due to an increased sensitivity to calcium, it may be counterbalanced by an increase in left ventricular wall stiffness, time to maximal contraction, and relaxation time (79, 80). Within the context of these myocardial changes, the increased contractility may be most effective at the slower heart rate and chronotropic effect of vasoactive medications may not be beneficial but detrimental. Alpha-adrenergic sensitivity may increase during hypothermia, but β_1_ adrenergic responses are variable during TH depending on the experimental model and temperatures investigated. Furthermore, TH also slows the oxygen consumption of the tissues and affects the drug metabolism (54). The Pharmacool study is currently evaluating the effects of therapeutic hypothermia on a myriad of drugs used for the asphyxiated neonatal population, including antihypotensive medications (81). During rewarming, CO and systolic BP will increase while the simultaneous decrease in systemic vascular resistance will be reflected in a decreased diastolic and mean BP (82).

Dopamine, dobutamine, and epinephrine are frequently administered to neonates and the use of milrinone, norepinephrine and vasopressin has increased in recent years. Of note, fluid bolus is commonly given to hypotensive neonates first, followed by cardiovascular supportive treatment. However, the administration of fluid bolus may be harmful in asphyxiated neonates (83). Conversely, in neonates with hemorrhagic hypovolemia, such as asphyxia associated with placental abruption, tempered fluid or blood administration may be needed for proper hemodynamic responses toward inotropes or vasopressors. While hypoxia-reoxygenation injury can increase the permeability of the peripheral microvasculature and potentially exacerbate fluid shifts, TH may at least in part counteract these effects (84). Therefore, the intravascular volume status must be carefully assessed prior to fluid therapy (85, 86).

## Catecholamines

Catecholamines including dopamine, dobutamine, epinephrine and norepinephrine have a catechol and amine group in their chemical structure. Through the activation of adrenergic (and/or dopaminergic and serotoninergic) receptors in the sympathetic nervous system, cardiomyocytes, vascular smooth muscle cells, and other extravascular parenchymal cells, catecholamines produce complex cardiovascular, renal and endocrine actions (87). However, the expression of cardiovascular adrenoreceptors can be altered in critical illness, prolonged use of catecholamine, and relative adrenal insufficiency, in addition to immaturity and dysmaturity (88). The β-1 adrenoreceptors could be down-regulated, β-2 adrenoreceptors uncoupled from adenylyl cyclase, and adenylyl cyclase and cAMP production decreased in the failing myocardium (89).

## Dopamine

Dopamine is the most commonly used antihypotensive infusion used in neonates, due to the frequent use in the preterm population (90). Dopamine, an endogenous catecholamine precursor of norepinephrine, has sympathetic and neuro-endocrine properties. Its pharmacological actions are through the direct stimulation of dopaminergic and adrenergic receptors or indirect stimulation of dopamine_2_ receptor with release of norepinephrine stored in the peripheral sympathetic nerve endings (89, 91). Given the finite norepinephrine stores of neonates, dopamine may lose its effectiveness over time as the indirect inotropic pathway become depleted. Thus, dopamine should not be chosen for long-term inotropic therapy (92).

There have been extensive discussion regarding effects of dopamine at different doses, different gestations and ages (93). The controversy is the dose-dependent stimulation of dopaminergic and adrenergic receptors at “low” (<2–4 mcg/kg/min), “moderate” (4–10 mcg/kg/min), and “high” (10–20 mcg/kg/min) doses with “renal,” chronotropic/inotropic and vasoconstrictive effects, respectively, in neonates (94–96). There is little information available regarding the expression and functionality of adrenergic and dopaminergic receptors expressed in the cardiovascular system including renal, mesenteric, and coronary vasculatures, in asphyxiated neonates. The response to dopamine has a high interpatient variability, which is in part related to the overlapping stimulation of dopaminergic and adrenergic receptors, and can be markedly decreased in ill neonates (97, 98). Padbury et al. studied pharmacokinetics of dopamine in critically ill neonates with different pathophysiological conditions, including 7/13 with a diagnosis of asphyxia, and observed variable responses to the same dose in different patients (99). Thus, the dosage of dopamine should be titrated according to the hemodynamic effect but not standardized dosing regimen.

Much of our evidence was inferred from findings from animal studies and a small number of clinical trials in the neonatal population. There is a lack of consistent effect of dopamine on CO, BP, and organ perfusion in animal studies (33, 37, 49, 50). Based on our search, we found only one randomized controlled study that compared dopamine (2.5 mcg/kg/min) to placebo in asphyxiated term neonates (11). Despite the pressor effect after dopamine infusion, echocardiographic indices of CO, morbidity or mortality were not different between groups. In a study of 11 asphyxiated neonates, although CO, stroke volume, BP and heart rate were increased after dopamine infusion (4–10 mcg/kg/min, *n* = 6), no temporal hemodynamics were provided for comparison in 5 untreated infants (12). There is insufficient evidence to support using dopamine to improve outcome, morbidity and mortality in term infants with suspected asphyxia (100). Interestingly, Drury et al. demonstrated that dopamine infusion (4–40 mcg/kg/min titrated according to mean arterial BP) delayed but did not prevent terminal hypotension after severe asphyxia in near-term fetal sheep [517 (range 240–715) vs. 106 (range 23–497) min of saline-treated controls after the start of infusions] (47). Furthermore, there might be an adverse effect with the use of high dose dopamine. Increased PAP/SAP ratio leading to right to left ductal shunting with potentially aggravating hypoxemia has been observed in asphyxiated newborn piglets and premature infants (33, 37, 101). Compared to higher dopamine doses, the addition of epinephrine to dopamine had a more favorable lower PAP/SAP ratio in an asphyxia-reoxygenation piglet model (48).

Although a piglet model demonstrated no net change in myocardial oxygen delivery, a study in neonates after their Norwood surgery demonstrated that myocardial oxygen use was greater than delivery after a dose of 5 mcg/kg/min (16, 33). The regional hemodynamic effects of dopamine in asphyxiated newborns have been uncertain although there was increased mesenteric blood flow at high dose (>10 mcg/kg/min) in newborn piglets with asphyxia. The vasodilatory action of dopamine in the mesenteric vasculature suggests the activation of β-2 adrenergic (unlikely dopaminergic) receptors at high doses due to sub-optimal or impaired receptor functionality under hypoxia-reoxygenation. Further, it remains controversial if dopamine affects cerebral autoregulation (50, 102).

Although dopamine may cause chronotropy with tachycardia which may increase CO, its inotropic effect remains to be determined. We consider dopamine predominantly a vasopressive drug which could be considered for the treatment of low BP in the presence of a normal CO state. Indeed, there should be consideration of effects and interpatient variability at different doses and an understanding that high doses may increase the ventricular afterload and potentiate right to left shunting across the ductus arteriosus. Nevertheless, despite its fast onset of action and user familiarity, the lack of reliable inotropy in the face of potential excessive vasoconstriction and its ability to potentiate PHT do not make it an attractive first choice as the cardiovascular supportive agent for asphyxiated neonates.

## Dobutamine

Dobutamine is a synthetic catecholamine that has direct adrenoreceptor agonism. It acts predominantly on β-1 adrenoreceptors to increase cardiac contractility with improved ejection times and shortening fraction and often will increase heart rate, especially at mid to higher doses (103). There is little peripheral vasoconstriction and potentially mild peripheral vasodilation and afterload reduction (34, 103). A recent literature review on the pharmacology of dobutamine indicated that heart rates in neonatal studies increased an average of 7–15 beats/min (or 5–12% change) with the average dose of approximately 5 mcg/kg/min mitigating a significant heart rate change (103). At doses with associated tachycardia or >10% of the baseline heart rate, dobutamine may increase myocardial oxygen consumption which renders its effect on CO less efficient and potentially aggravates cardiac dysfunction in the presence of unmatched oxygen delivery. As dobutamine acts directly as a catecholamine, long-term use will result in tachyphylactic down regulation of catecholamine receptors.

While dobutamine is not a common medication used in neonates with isolated hypotension, it is often used in asphyxiated neonates with low CO or perfusion states with or without hypotension (90, 104). Two small studies of 6 term asphyxiated neonates and 13 critically ill neonates respectively, demonstrated that dobutamine at 2.5–10 mcg/kg/min increased CO and heart rate but not necessarily arterial BP (13, 105). Interestingly, dobutamine has been shown to be more effective at increasing CO during rewarming (35–37°C) than during cooling temperatures (33°C) in a small study of neonates receiving TH for asphyxia (78). This temperature dependence has also been demonstrated in ventricular trabeculae tissue studies and may be further elucidated with the Pharmacool results (106).

While neonatal animal studies of dobutamine during normoxia demonstrated a dose-dependent increase in CO, through both chronotropy and inotropy, with a mild increase in cerebral flow, an improved CO was replicated only at 20 mcg/kg/min without significant increases in regional perfusion in an asphyxia-reoxygenation piglet model (35, 36, 38). A recent asphyxia-reoxygenation piglet study affiliated with the NeoCirc Trial utilized a new dobutamine formulation and demonstrated increases in CO, in the face of unchanged stroke volume, oxygen delivery, and oxygen consumption with improved peripheral tissue oxygenation index and peripheral intravascular oxygenation at doses of 10–15 mcg/kg/min (51).

Dobutamine, with inotropic and mild afterload reduction properties, has a relatively quick onset of action (minutes) in comparison to milrinone (hours), another drug with similar properties; thus, is a reasonable first line therapy for asphyxiated babies who have clinical or echocardiographic decreases in CO and increased systemic vascular resistance or maintained BP pressure. Dobutamine does not have the significant metabolic effects of hyperglycemia and hyperlactatemia that can be seen with epinephrine. Therefore, for those clinicians who use serial lactate measurement as a sign of improvement in tissue perfusion, the inotropy of dobutamine is not accompanied by an increase in lactate to the same magnitude of the inotropic dose of epinephrine. However, the lack of significant vasopressive action of dobutamine cautions its use, as a single agent, in certain asphyxiated neonates with normal or borderline BP as the BP could further be reduced. Indeed, if vasopressive action is required during dobutamine infusion, the addition of dopamine, norepinephrine or vasopressin may be considered (4). Further the use of dobutamine should be cautious in neonates with preexisting tachycardia or ventricular outflow tract obstruction (e.g., asphyxia in an infant of diabetic mother or with hypertrophic cardiomyopathy) because of the aggravated β-1 adrenoreceptors agonism.

## Epinephrine

Epinephrine is an endogenous catecholamine and it stimulates both α (-1 and−2) and β (-1 and−2) adrenoreceptors, which is believed to have dose-dependent hemodynamic effects. In neonates, at low doses (0.02–0.1 mcg/kg/min) epinephrine acts predominantly on the β-1 and β-2 adrenoreceptors and has positive inotropic and chronotropic actions (97). We also demonstrated limited vasodilation in pulmonary, renal and mesenteric vasculatures of asphyxiated-reoxygenated newborn piglets (42). Similar observations in the systemic and pulmonary hemodynamics with improved CO, increased BP and lowered PAP/SAP ratio (favorable reversal of right-to-left ductal shunting in PHT), were found in studies using newborn animals (33, 39, 40). At doses >0.1 mcg/kg/min, the α-1 adrenoreceptor effect of vasoconstriction predominates over the β adrenergic effect resulting in decreasing blood flow to the gut and kidneys. This may further increase the afterload of an already compromised neonatal heart and tissue perfusion. Indeed, high dose epinephrine (>0.5 mcg/kg/min, equivalence of a resuscitation dose of 10 mcg/kg every 20 min) may lead to decreasing CO due to coronary vasoconstriction and disorganized energy utilization.

Epinephrine infusions are commonly associated with hyperglycemia and hyperlactatemia due to increased glycogenolysis via the activation of β-2 adrenergic receptors. This may confound the use and interpretation of lactate during epinephrine infusion as lactate is generally known as a marker of the anaerobic glycolytic breakdown of glucose to pyruvate exaggerated by α-adrenergic vasoconstriction and imbalanced oxygen metabolism vs. as a byproduct of accelerated aerobic glycolysis (27). Studies indicate that increases in glucose and lactate were related to increasing doses of epinephrine infusion but not associated to age, birthweight or glucose infusion rate and were often delayed in comparison to elevation in BP or heart rate (27). Associated hyperglycemia may necessitate insulin infusion and the need for more vascular access points if infusion capability issues arise. Interestingly, Basu et al. found that while hypoglycemia and hyperglycemia were independently associated with death and/or severe neurodevelopmental disability at 18 months in infants with hypoxic-ischemic encephalopathy, they further observed that hyperglycemia in those infants randomized to TH had reduced risk of unfavorable outcome in a *post-hoc* analysis of 234 infants enrolled in the CoolCap Study from 1999 to 2002 (ADC 2016 and ADC 2017) (107, 108). This high-lights the importance of glucose homeostasis and its cerebral consequences and subsequent neurodevelopmental outcome. In the choice and administration of inotropes in asphyxiated neonates, euglycemia needs to be ensured. Other adverse cellular effects with epinephrine infusion have been reported including myocardial oxidative stress, sarcolemma rupture and increased cytoplasmic calcium deposits (32, 40).

Epinephrine is considered for hypotensive asphyxiated neonates when there is a concern of decreased CO with decreased systemic vascular resistance due to vasoplegia. Given the increases in heart rate and myocardial contractility, clinicians should ensure that volume status is adequate prior to starting epinephrine. It is also cautious to use epinephrine in the face of ventricular outflow tract obstruction. Clinicians need to be cognizant of the metabolic increases in lactate as it may change their ability to follow serial lactates for signs of hemodynamic or oxygen utilization improvement. Interestingly, Manouchehri et al. demonstrated that the use of epinephrine in combination with dopamine improved systemic and regional hemodynamic effects with reduced PAP/SAP ratio, which was deficient in high-dose dopamine infusion (48). Further studies are needed to evaluate the innovative proposal of starting epinephrine infusion at low doses (0.05~0.1 mcg/kg/min) as the initial choice of a mono-pharmacological, cardiovascular supportive therapy in those asphyxiated hypotensive neonates with evidence of low CO and poor vessel tone.

## Milrinone

Milrinone is a bipyridine derivative that inhibits phosphodiesterase type III on cAMP degradation leading to increased intracellular calcium concentration (52). It is named as an inodilator because of intracellular calcemic actions in cardiac myocytes (improved contractility) and vascular smooth cells (vasodilation, both systemic and pulmonary). Milrinone increases myocardial contractility through a cyclic AMP-mediated increase in trans-sarcolemmal calcium flux and also allows calcium resequestration into the sarcoplasma reticulum, causing peripheral vasodilation, and improved actin-myosin dissociation during diastole; thus, earning its classification as an inotrope and lusitrope (109). Bianchi et al. demonstrated significant increases in CO within 6 h of milrinone infusion in a prospective study of preoperative neonates with congenital heart defects at an average age of 12 h of life (28). Milrinone may have the advantage over some other inotropes regarding a possible preservation of myocardial contractile response with its ongoing use for the post-adrenoreceptor effects, which also explains its gradual onset of action (25, 89, 110). Tachycardia, hypotension, and thrombocytopenia have been reported as side effects, with transient hypotension occurring more frequently in the setting of compromised vascular volume and/or bolus loading doses (111, 112).

The combined inotropic and pulmonary vasodilatory effects of milrinone, as facilitated by its lusitrophic action on diastolic dysfunction, is advantageous in asphyxiated neonates with impaired myocardial contractile function and PHT, resulting in improved CO and oxygenation. Interestingly, milrinone has been shown to dilate the ductus arteriosus in rats because of the phosphodiesterase type III inhibition (43). These hemodynamic actions may prove to be important in the abnormal cardiovascular state of asphyxiated neonates and make milrinone a potential medication for asphyxia with PHT (113).

The half-life of milrinone is approximately 4 h in neonates who have decreased clearance (32, 109). While the renal clearance of milrinone depends on gestational and chronological age (up to 10 days) in term neonates, dose adjustments in the presence of significant renal impairment, prematurity, or in the first few days after birth may be warranted to avoid an accumulation of dose and accompanying side effects (30).

Over the last decade there has been an increasing use of milrinone in neonates predominantly for cardiopulmonary dysfunction in the context of PHT and low CO in diseases including asphyxia, peri-operative period of cardiac surgery, and congenital diaphragmatic hernia (14, 15, 17, 28, 111, 114–116). In newborn piglets with asphyxia, dose-related increases in systemic and regional (carotid and intestinal) hemodynamics without significant effects on chronotropy, BP and PHT were observed with milrinone infusion at 0.25–0.75 mcg/kg/min (41). Despite a lack of quality data found by a 2015 Cochrane review to recommend its use for PHT in neonates, small feasibility studies have shown milrinone increased performances of both left and right ventricles, and improved oxygenation (29, 112, 114, 116, 117).

Although milrinone is emerging as an attractive therapy in neonates with cardiovascular dysfunction compromised with PHT, clinical studies of high quality are mandatory to examine its therapeutic and prophylactic uses in asphyxiated neonates with PHT who have or may develop hypotension and low CO and poor tissue perfusion (118–120).^.^

## Norepinephrine

Norepinephrine is an endogenous sympathomimetic amine that acts primarily on vascular and myocardial α-1 receptors with a mild β-1 and minimal β-2 adrenoreceptors stimulation. Therefore, norepinephrine has predominant peripheral vasoconstriction with minimal inotropic effect (121). Indeed, the concern of myocardial vasoconstriction and oxygen delivery as well as the afterload increase in the presence of myocardial dysfunction may preclude the use of norepinephrine as the first drug of choice in asphyxiated neonates with suspected increased or normal vascular tone.

Norepinephrine's ability to increase systolic pressure also improves coronary systolic BP which is important to the coronary blood flow of the right ventricle especially during acute right heart failure as can be seen when exposed to the high afterload of PHT (122, 123). Thus, in addition to increasing BP, norepinephrine may assist in decreasing pulmonary hypertension while improving cardiac performance of the left and right ventricle. However, on a cautious note, excessive systemic vasoconstriction from norepinephrine infusion can further impair ventricular contractility, in addition to increased myocardial oxygen demand, when ventricular function is not concurrently supported.

To date, the clinical use of norepinephrine in term neonates has been predominantly reserved for hypotensive neonates who have refractory shock or low CO state, especially those with severe septicemia and post cardiac surgery or right ventricular stress (19). In 22 term neonates with refractory septic shock and receiving moderate to high doses of dopamine or dobutamine, norepinephrine significantly increased mean BP (36 ± 5 to 51 ± 7 mmHg), improved oxygenation measures and decreased serum lactate within hours (20). The increase in oxygenation could be related to differential effects of norepinephrine at the systemic and pulmonary vasculature resulting in a favorable increase in systemic-to-pulmonary arterial pressure ratio.

Oualha et al. reported pharmacokinetic and pharmacodynamic variabilities during the infusion of norepinephrine (0.5–3 mcg/kg/min) in 11 neonates (124). They suggested that birthweight, age and severity of illness might affect the production and clearance of norepinephrine, which are dependent on enzymatic maturation. The mean doses used in this study were on the high end of dosing regimens in comparison to pediatric or adult doses used in current practice and judicious dosing commenced earlier in shock courses may allow for lower doses or earlier discontinuation of other inotropes as seen in retrospective reviews (125).

Norepinephrine is often used as a second or third line antihypotensive drug. In combination with dobutamine or milrinone, it allows for vascular tone support, and may augment coronary perfusion and support the right ventricle myocardium in the case of asphyxia with severe PHT and right heart failure (123). The addition of norepinephrine 0.02–0.05 mcg/kg/min might reduce epinephrine dosages (leading to less hyperlactatemia and hyperglycemia) in asphyxiated neonates with severe cardiac dysfunction and vasoplegia. Further studies are required to evaluate the therapeutic role of this combined use of α and β adrenoreceptors agonists.

## Vasopressin

Vasopressin, or arginine vaspressin (AVP), is an endogenous substance with diverse actions that relate to the location of tissue specific vasopressin subtype receptors, but is most commonly used for its vasoconstrictor properties. The V1 subtype mediates vasoconstriction in vascular smooth muscle except within the pulmonary circulation where it potentiates the release of nitric oxide causing vasodilation. V1 receptors are also found on hepatocytes and platelets, which could result in platelet aggregation and glycogenolysis. The V2 subtype regulates water reabsorption, while V3 effects adrenocorticotrophic hormone (ACTH) release (126–128). Purinergic P2 receptors are also found in the myocardium and contribute to selective coronary dilation. During exogenous infusion, the potent vasoconstrictive effects predominate (129). In neonatal studies, dosages of AVP ranged between 0.0002–0.002 units/kg/min in keeping with manufacturer recommendations (130, 131).

Vasopressin, in the presence of ACTH, is associated with increased adrenal cortisol release (132). The use of vasopressin has been increasing over the past 10 years in neonates with sepsis, catecholamine and corticosteroid resistant shock, hypotension from ventricular outflow tract obstructions, post cardiopulmonary bypass for congenital cardiac surgery and PHT (18, 24, 130, 133, 134). It is speculated that endogenous AVP is depleted in these critical vasoplegic conditions (135, 136). Low-dose AVP infusion selectively dilated pulmonary, coronary, and even cerebral vasculature under hypoxic state, but vasoconstricted other vascular beds (31, 137–139). Cheung et al. demonstrated that vasopressin treatment causes a dose-dependent, baro-specific effect, while preserving cardiac function and cerebral and mesenteric hemodynamics in asphyxiated newborn piglets during the infusion of vasopressin at low doses (0.00008–0.0003 units/kg/min) (31). Pelletier et al. further showed the low-dose vasopressin infusion improved CO and mesenteric perfusion using the swine model of neonatal asphyxia with lowered oxidative stress markers similar to those observed in animals with dobutamine infusion at 20 mcg/kg/min (46). Retrospective studies also suggest a potential role for vasopressin in neonates with congenital diaphragmatic hernia or PHT with catecholamine resistant hypotension, when it was used as an adjunctive antihypotensive therapy at 0.0001–0.005 units/kg/min (26, 140). Improvements in mean arterial BP, oxygenation index, heart rate, pulmonary-to-systemic shunts, inotrope scores and the need for ECMO have been found.

However, despite increased BP, decreased inotropic support and variable effects in end-organ perfusion, clinical studies of vasopressin use did not demonstrate an increase in survival (126, 141). Given that vasopressin is often used as a rescue therapy for profoundly hypotensive neonates late in the shock process, this may not be surprising. Of note, in a pediatric randomized controlled trial that did not include neonates, a trend toward increased mortality was observed in children treated with low-dose vasopressin (142).

Adverse effects of vasopressin that have been reported in neonatal studies include significant hyponatremia, transient decrease in platelet count, and liver necrosis (24, 26, 133, 143). Although, limb necrosis has been reported in the meta-analysis and Cochrane review, these predominantly occurred with the use of terlipressin but Choong's pediatric randomized study reported an increase in the vasopressin group (26, 142).

The therapeutic role of vasopressin in asphyxiated neonates with profound vasoparesis and hypotension and/or PHT is unclear. Vigilance must be taken to watch for hyponatremia and evidence of worsening cardiac dysfunction in the face of vasoconstriction.

## Levosimendan

Levosimendan is a “calcium-sensitizing inotrope” that acts to increase cardiac contractility by binding to and stabilizing calcium-saturated cardiac Troponin C. This inotropic action of levosimendan differs from that of catecholamines or milrinone which increase the trans-sarcolemmal calcium flux during systole. Consequently, the increased CO by levosimendan is not associated with higher myocardial oxygen consumption, which is commonly seen with catecholamines administration (144). Levosimendan partially inhibits phosphodiesterase type III and activates ATP-sensitive potassium channels, resulting in mild lusitropy and vasodilation, respectively (thus an inodilator) (145–147). These additional properties of levosimendan may cause systemic and pulmonary vasodilation and increase coronary blood flow (144).

Levosimendan is mostly used in neonates and infants with heart failure or myocardial stunning (21–23, 148). However, the hemodynamic effects and dosing regimen of levosimendan was seldom described in detail in asphyxiated neonates. The limited literature and experience may therefore render the use of levosimendan mostly as an adjunctive cardiovascular supportive therapy of critically ill neonates in countries where its clinical application is approved. Using a swine model of neonatal asphyxia, we found that levosimendan at 0.1–0.2 mcg/kg/min had only a modest improvement in CO and carotid blood flow without significant hypotension, tachycardia, or worsening of PHT (44). This suggests that the calcium sensitizing action of levosimendan may not be effective in the neonatal myocardium after asphyxia, thus undermining its role as a monotherapy in asphyxiated neonates.

Given that cAMP-increasing inotropes increase intracellular calcium and that levosimendan sensitizes the myofibrils to calcium, it is speculated that the two classes may have a synergistic effect. Nevertheless, the therapeutic role for levosimendan in neonatal asphyxia deserves further investigations as an adjunctive therapy to minimize the dosage and side effects of catecholamines used.

## Hydrocortisone

An intact hypothalamo-pituitary-adrenal axis function is critical for shock reversal. Steroid has an important role in the function of adrenergic receptors with enhanced coupling and adenylate cyclase activity (149). Steroids may also up-regulate myocardial and vascular adrenoreceptors (150). Paired with their ability to increase circulating catecholamines by inhibiting degradation, steroids can augment catecholamine action. Smooth muscle calcium availability is also increased improving vessel tone and decreasing capillary leak.

In neonatal asphyxia, adrenal insufficiency and or adrenal hemorrhage has been reported (151). Indeed, adrenal hemorrhage may be found in neonates with severe asphyxia and those with poor outcome. In asphyxiated newborn piglets, Chapados et al. observed a delayed cortisol response to standard adrenocorticotrophin (ACTH, Cortrosyn 4 μg/kg) stimulation whereas the resuscitation with 100% but not 21% oxygen had a sub-optimal increase in cortisol levels (152). This suggests a possible reduction in the adrenal reserve and dysfunctional hypothalamo-pituitary-adrenal axis in the asphyxiated newborn piglets, especially when a high oxygen concentration was used in the resuscitation. They suggested that the preserved cortisol response in 21% oxygen resuscitation was related to the expression of steroidogenic factor 1 in the adrenal glands.

Although the clinical significance of cortisol response to ACTH in neonates is uncertain, a poor response to a standard ACTH stimulation test (an increase in plasma cortisol of <250 nmol/l, 1 h post injection) has been associated with higher short-term mortality in adults and children with septic shock (153, 154). Interestingly, low serum cortisol levels in critically ill term neonates who subsequently respond appropriately to exogenous ACTH may also indicate that some neonates have inadequate hypothalamic-pituitary-adrenal signaling with intact adrenal function (155). Thus, the *physiological* and *pharmacological* administration of steroids or hydrocortisone seems like a reasonable and trendy modality to provide cardiovascular support (156). Fernandez et al. showed that hypotensive infants harboring low cortisol levels (<414 nmol/l in stress) showed an improvement in their hemodynamic parameters when given hydrocortisone for their shock (1–2 mg/kg/day) compared to infants with normal cortisol levels (155). It indicates that hydrocortisone administration to reverse (catecholamine-) refractory shock can be beneficial only in those with an impaired adrenal function. However, there is limited information regarding the therapeutic roles of hydrocortisone therapy (adrenal dysfunction modulation and or anti-inflammatory action) in asphyxiated newborns (151). Furthermore, clinicians should attend to the potential side effects of hydrocortisone including hyperglycemia, gastrointestinal hemorrhage, and increased pulmonary arterial pressure for short-term administration of hydrocortisone (157).

## Final thoughts

**Figure 1:**
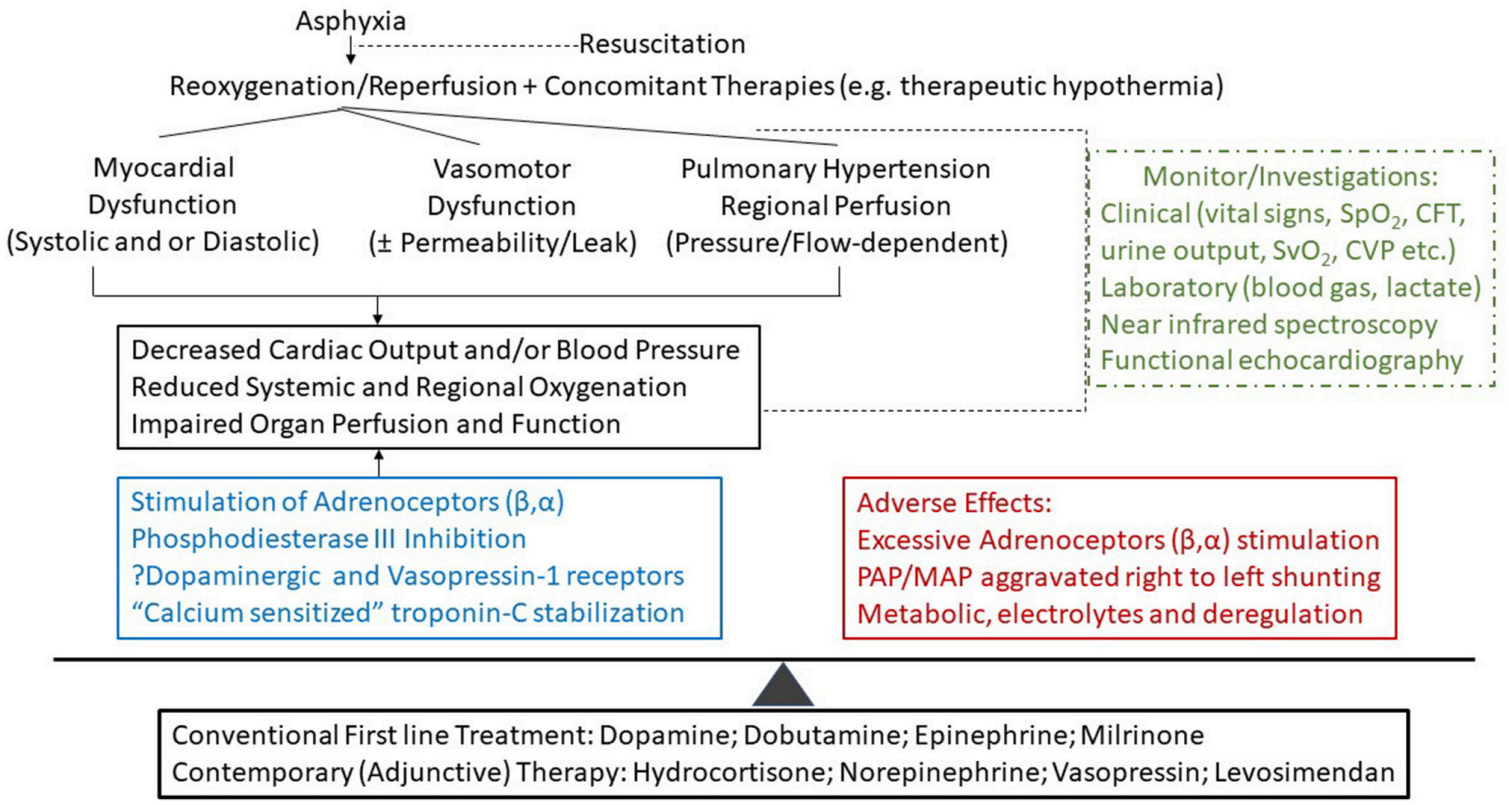
A proposal of therapeutic approach in the cardiovascular support of asphyxiated neonates. The choice of cardiovascular medication(s) depends on the targeted approach for cardiac output and blood pressure management with improved oxygenation, organ perfusion and function which need to be balanced against the adverse effects of therapies. CFT, capillary filling time; CVP, central venous pressure; SpO_2_ and SvO_2_, percutaneous (arterial) and venous oxygen saturation, respectively.

Asphyxiated neonates have unique pathophysiological and pharmacological conditions for shock with regional hypoperfusion which interplay with the hemodynamic effects of TH, respiratory state and ventilatory support. In addition to clinical assessment, application of evaluating tools of cardiac function, and tissue oxygenation will enhance our understanding of the pathophysiology of hypotension and /or low CO in these critically ill neonates. Therefore, individualized cardiovascular supportive therapy can be provided based on pharmacological and patho-physiological understanding of the neonate, therapy, and concurrent interventions accordingly (Figure 1)

**Figure 2:**
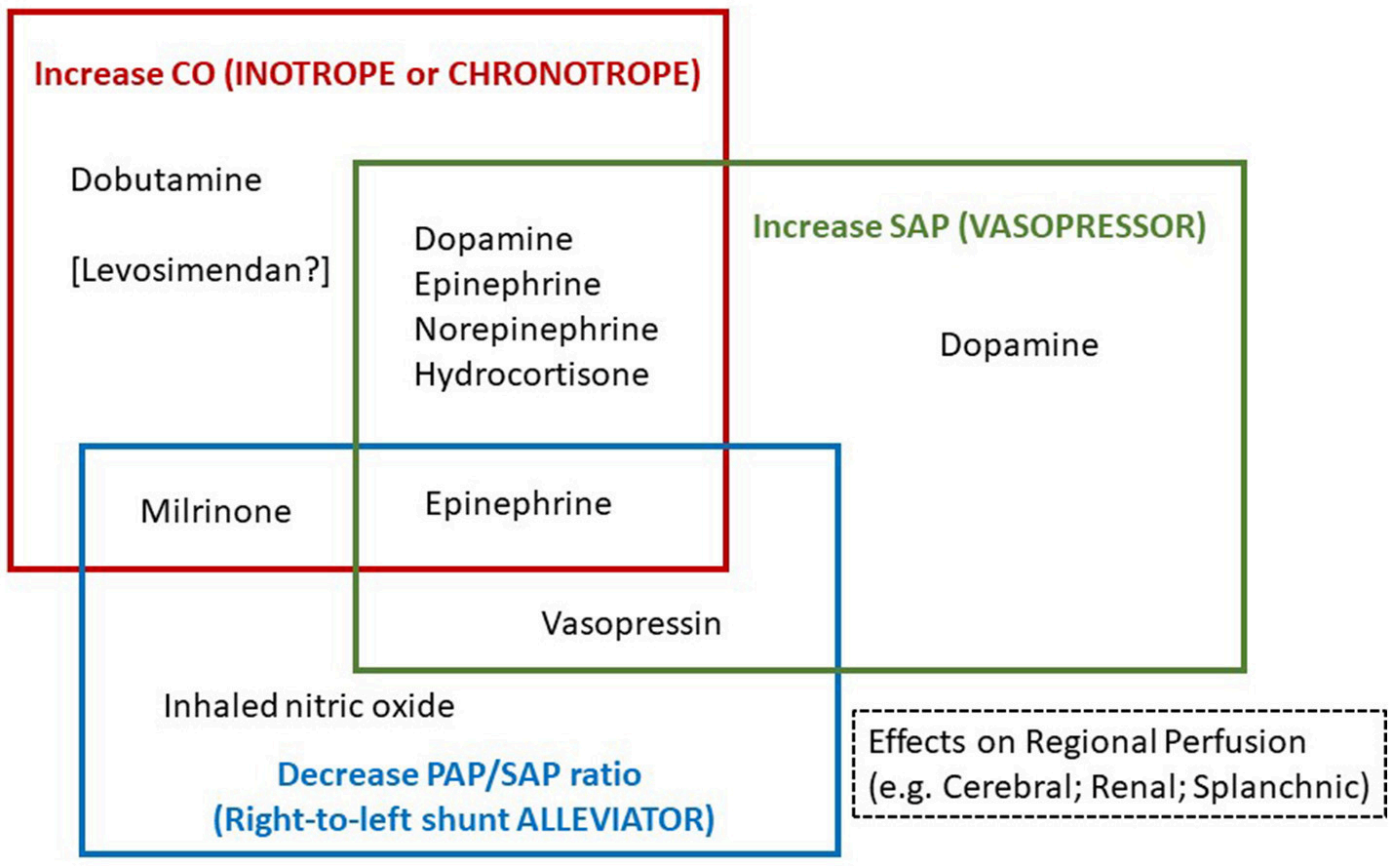
Medications categorized according to three specific cardiovascular supportive functions; (a) to increase cardiac output (CO), (b) to increase systemic arterial pressure (SAP), or (c) to decrease pulmonary arterial pressure (PAP) to SAP ratio.

In this review, the asphyxiated neonate develops “hypotension” and/or “low CO” which we refer to clinical condition(s) with tissue perfusion deficits as a result of low BP and or decreased systemic perfusion, respectively. The hypotensive and low CO states therefore require respective therapies. Despite the common occurrence of hypotension and low CO in asphyxia, some neonates have hypotension with normal CO state or reduced systemic blood flow without severe hypotension (4). The cardiovascular supportive therapies are directed toward the correction of specific perfusion deficits (Figure 2). In view of potential adverse effects of cardiovascular supportive therapy, especially at high dosages, the lowest effective dosage of medications should be used to provide *optimal* BP for adequate organ (e.g., cerebral or renal) perfusion and systemic oxygenation, and *optimal* CO without tissue perfusion deficits and metabolic acidosis. Furthermore, the regional hemodynamic effects of medications also play an important role in the choice of cardiovascular supportive therapies.

We identified pre-clinical studies but very few clinical studies conducted to examine systemic and regional hemodynamic effects of various cardiovascular supportive medications in this critical population. While the interpretation and translation of the findings from animal studies require great caution, the studies provide important information that may help tailor appropriate medical cardiovascular support that maintains BP, CO, and regional perfusion. It is imperative to note that, treating hypotension, as opposed to poor CO or decreased blood flow, has not convincingly improved mortality and morbidity (93, 96, 158). Targeting CO and regional perfusion may be the appropriate approach for improving the outcome of these critically ill neonates with asphyxia. The resultant cardiovascular effects of medications should be balanced against adverse effects including excessive vasoconstriction, tachycardia, and metabolic effects associated with the collateral receptor(s) activation at high doses. It is also important to attend to the metabolic effects of inotropes, e.g., hyperglycemia and hyperlactatemia of epinephrine infusion given euglycemia and normal lactate levels are critical for clinical care and predictive for neurodevelopmental outcomes.

The information regarding the co-administration of catecholamines is scare. The choice of catecholamines will depend on the synergetic activation of adrenoceptors and signaling pathway in myocardial contractile function, systemic and pulmonary vasomotor regulation, potentially without excessive adrenoceptors activation of high-dose monotherapy. Pharmacodynamic and pharmacokinetic interactions in the neonatal cardiovascular system of combined therapies remain to be further examined.

## Author Contributions

CJ and P-YC both made substantial contributions to the conception or design of the work, and literature review. CJ drafted the work. CJ and P-YC revised it critically for important intellectual content and approved it for publication. CJ and P-YC agreed to be accountable for all aspects of the work in ensuring that questions related to the accuracy or integrity of any part of the work are appropriately investigated and resolved.

### Conflict of interest statement

The authors declare that the research was conducted in the absence of any commercial or financial relationships that could be construed as a potential conflict of interest.

## Footnotes

^1^We refer “hypotension” and “low cardiac output” to clinical condition(s) that the asphyxiated neonate has low blood pressure (leading to e.g., cerebral or renal perfusion deficits, right-to-left shunting across patent ductus arteriosus) and decreased systemic perfusion deficits (e.g., tissue hypoxia), and therefore require respective therapies.
